# Non-target ROIMCR LC–MS analysis of the disruptive effects of TBT over time on the lipidomics of *Daphnia magna*

**DOI:** 10.1007/s11306-023-02030-w

**Published:** 2023-08-07

**Authors:** Jamile Mohammad Jafari, Josefina Casas, Carlos Barata, Hamid Abdollahi, Romà Tauler

**Affiliations:** 1 Institute for Advanced Studies in Basic Sciences (IASBS), Zanjan, Iran; 2 RUBAM, Institute for Advanced Chemistry (IQAC-CSIC), Jordi Girona 18-26, 08034 Barcelona, Spain; 3 Liver and Digestive Diseases Networking Biomedical Research Center (CIBEREHD), Instituto de Salud Carlos III, 28029 Madrid, Spain; 4 Department of Environmental Chemistry, Institute of Environmental Assessment and Water Research (IDAEA), Spanish Research Council (IDAEA-CSIC), Barcelona, Spain

**Keywords:** Non-targeted lipidomics, ROIMCR, Three-way data analysis, Trilinear and bilinear modelling

## Abstract

**Introduction:**

This study has investigated the temporal disruptive effects of tributyltin (TBT) on lipid homeostasis in *Daphnia magna*. To achieve this, the study used Liquid Chromatography–Mass Spectrometry (LC–MS) analysis to analyze biological samples of *Daphnia magna* treated with TBT over time. The resulting data sets were multivariate and three-way, and were modeled using bilinear and trilinear non-negative factor decomposition chemometric methods. These methods allowed for the identification of specific patterns in the data and provided insight into the effects of TBT on lipid homeostasis in *Daphnia magna*.

**Objectives:**

Investigation of how are the changes in the lipid concentrations of *Daphnia magna* pools when they were exposed with TBT and over time using non-targeted LC–MS and advanced chemometric analysis.

**Methods:**

The simultaneous analysis of LC–MS data sets of *Daphnia magna* samples under different experimental conditions (TBT dose and time) were analyzed using the ROIMCR method, which allows the resolution of the elution and mass spectra profiles of a large number of endogenous lipids. Changes obtained in the peak areas of the elution profiles of these lipids caused by the dose of TBT treatment and the time after its exposure are analyzed by principal component analysis, multivariate curve resolution-alternative least square, two-way ANOVA and ANOVA-simultaneous component analysis.

**Results:**

87 lipids were identified. Some of these lipids are proposed as *Daphnia magna* lipidomic biomarkers of the effects produced by the two considered factors (time and dose) and by their interaction. A reproducible multiplicative effect between these two factors is confirmed and the optimal approach to model this dataset resulted to be the application of the trilinear factor decomposition model.

**Conclusion:**

The proposed non-targeted LC–MS lipidomics approach resulted to be a powerful tool to investigate the effects of the two factors on the *Daphnia magna* lipidome using chemometric methods based on bilinear and trilinear factor decomposition models, according to the type of interaction between the design factors.

**Supplementary Information:**

The online version contains supplementary material available at 10.1007/s11306-023-02030-w.

## Introduction

Every year, new chemicals and toxic compounds (biocides, pesticides, metal alloys, drugs, etc.) are introduced to the market and subsequently into the environment. Among the known toxic compounds, organotin compounds (OTCs) are environmental contaminants known to be persistent, toxic, and bio-accumulative. They belong to organometallic compounds used for agricultural, industrial, and biomedicinal applications (Karlaganis et al., 2001; Lyssimachou et al., 2009; Wong et al., 2012). Tributyltin (TBT), one of the most harmful OTCs, is widely recognized to have a biological effect on the hormone system (Graceli et al., 2013), acting as an endocrine-disrupting chemicals (EDC). Endocrine disruption, frequently linked to widespread metabolic changes, spans from aquatic to terrestrial organisms and is extensively studied for its profound biological and ecological effects (Pagliarani et al., 2013).

The toxicity of a compound is a complex phenomenon that is influenced by a multitude of factors, including its concentration, duration of exposure, bioavailability, and the sensitivity of the biota. Other physical factors such as temperature and salinity can also play a role in determining the toxicity of a compound, and the presence of other compounds in the environment can lead to interactions that further complicate the assessment of toxicity. To accurately assess toxicity, it is important to consider all of these factors, and this often requires conducting multiple experiments with different treatment and control groups (Jansen et al., 2005). This results in multivariate and multiset data sets that are characterized by various sources of variation (Smilde et al., 2005). Effective data analysis techniques are needed to understand the underlying systems and relationships between the variables.

In multivariate omics studies, altering two factors using an appropriate experimental design can produce three-way data sets, which have three data modes, ways or directions representing the values of different measured variables under two or more factors at different levels. Analyzing this type of data requires careful consideration of the relationships between the structure of the data and the model used for their analysis, as well as of the effects of the experimental design factors. Different types of interactions between factors can occur, such as additive, multiplicative, synergistic, antagonistic, (Bansal et al., 2013; Blair & Taylor, 1997; Coors & De Meester, 2008; Taylor, 1989), and randomly and it is important to identify the type of interaction between the investigated factors in order to analyze omics data effectively.

Lipidomics is a branch of metabolomics that focuses on the comprehensive study of lipid species and their associated networks and metabolomic pathways of a biological system. Targeted and non-targeted analytical procedures are the two main types of lipidomic studies (Lam & Shui, 2013; Sethi & Brietzke, 2017). The targeted strategy concentrates on examining a particular list of lipids, usually associated with a known metabolic pathway of interest, to confirm an a priori hypothesis. In contrast, non-targeted lipidomics aims to globally analyze all measurable lipids present in a sample without any prior assumptions about affected pathways or lipid species. (Cajka & Fiehn, 2014; Fenaille et al., 2017; Navarro-Reig et al., 2018; Navas-Iglesias et al., 2009; Wolf & Quinn, 2008; Xu et al., 2020; Aldana et al., 2020; Lippa et al., 2022). Effective lipidomics analysis can provide valuable insights into the metabolic pathways involved in lipid metabolism, and help in the identification of potential biomarkers for disease diagnosis and therapy.

Chemometrics provides useful tools for studying chemical systems, and a new methodology has been proposed that combines the region of interest (ROI) concept (Bedia et al., 2016; Gorrochategui et al., 2015, 2019; Tautenhahn et al., 2008) with the Multivariate Curve Resolution Alternative Least Square (MCR-ALS) method (De Juan et al., 2014; Jaumot et al., 2015; Tauler, 1995) for filtering, compressing, and resolving large data sets collected in LC–MS analysis of metabolomics data (Gómez-Canela et al., 2018; Gorrochategui et al., 2019; Pérez-Cova et al., 2021). The ROI method allows for significant reduction and filtering of raw MS data without decreasing mass accuracy, and can easily compress large amounts of data. This methodology has been successfully applied in several studies on the effects of chemical contaminants on aquatic organisms and their metabolic pathways (Gorrochategui et al., 2019; Perez-Lopez et al., 2021).

The process of selecting Regions of Interest (ROI) in LC–MS raw data has been previously described in existing literature referenced in the paper (Tautenhahn et al., 2008; Gorrochategui et al., 2016, 2019; Dalmau et al., 2018, Perez-Cova et al. 2021, Perez-Lopez et al., 2021). The ROI methodology aims to identify and select important HRMS (High-Resolution Mass Spectrometry) signals from each sample’s analysis, forming corresponding MSROI data matrices. The ROI method involves scanning mass spectra to identify regions where: (a) the ion signal exceeds a specified threshold above instrumental noise; (b) the masses align within a predetermined mass accuracy tolerance; and (c) the masses occur consistently over a time range that aligns with the expected width of a chromatographic peak. This selected time range is determined based on the instrument setup (e.g., gas or liquid chromatography) and the specific chromatographic column being used. Implementing the ROI methodology significantly reduces the amount of computer storage and resources required while maintaining the mass accuracy of the original HRMS measurements. This approach defines the signals that should be considered in subsequent data analysis and resolution. The number of ROIs should encompass all significant features relevant to the sample’s composition.

In the present study, the ROIMCR modeling of ROI LC–MS data is used for the analysis, resolution, relative quantitation, and identification of lipids of *Daphnia magna* samples under the exposure of tributyltin TBT over time. Relationships between the effects of two design factors (TBT exposure and time), their interaction, the three-way data structure and the more adequate model to be used are investigated. The freshwater crustacean ecotoxicological model D. magna was chosen for this study since it is among the most widely used organisms in aquatic environmental risk assessment of chemicals (Baird & Barata, 1998). D. magna is also known to accumulate high concentrations of lipids from its food during a reproductive or intermolt cycle to be allocated to the formation of the new carapace or eggs (Jordão et al., 2015). This means than these organisms are a good model to study contaminant effects on lipid homeostasis (Jordaõ et al., 2016).

This paper builds on two previous works (Jafari et al., 2020; Malik et al., 2016) that studied the effects of TBT exposure and time on the lipidomics of *Daphnia magna*. The first study used a targeted analysis approach with MCR-ALS, while the second study simulated scenarios to investigate the structure of lipidomics data under different effects of the two factors. This paper applies the conclusions of the second study to a new non-targeted analysis approach using the ROIMCR method, aiming to discover what were the lipids associated with changes in TBT levels and time and to determine if the structure of the data follows a trilinear model, indicating a synergistically multiplicative interaction between the factors.

## Materials and methods

### Experimental design and data acquisition

The study of the effects of TBT treatment on the dynamics of lipids across an entire adolescent inter-molt cycle of *Daphnia magna* (Clone F) was performed at two TBT doses: 0.1 µg/L (L), and 1.0 µg/L (H). These concentrations were selected from previous work to cause low and high effects on the accumulation of lipids (Jordaõ et al., 2016; Jordão et al., 2015). Experiments were performed at high food levels in adolescent females (5 × 10^5^ cells/mL of *Chlorella Vulgaris*) and contained five samplings that cover the whole intermolt/egg provisioning period: 0 h (just after the third molt), 8 h, 16 h, 24 h, and 48 h (just after the fourth molt and release the first brood of eggs into the brood pouch). Three replicates of 5 individuals from each sampling were taken and processed for total lipid analysis. For the 48 h period debrooded females were used. Two different independent but consecutive experiments were conducted because a large number of synchronized animals were required. Each replicate consisted of a pool of 5 animals that were homogenized in 500 ml phosphate buffered saline (PBS) pH 7.4 with 2,6-di-tert-butyl-4-methylphenol (BHT) 0.01%, as an antioxidant. The liquid chromatography-mass spectrometer consisted of a Waters Aquity UPLC system connected to a Waters LCT Premier Orthogonal Accelerated Time of Flight Mass Spectrometer (Waters, Millford, MA), operated in positive and negative electrospray ionization mode. Full scan spectra from 50 to 1500 Da were obtained. Mass accuracy and reproducibility were maintained by using an independent reference spray via Lock-Spray. More details on the experiments and analysis can be found elsewhere (Jordão et al., 2015; Gorrochategui et al., 2014).

### ROIMCR data analysis

This study employed a comprehensive untargeted lipidomics analysis approach to investigate the large number of lipid signals present in the samples. The study did not rely on prior knowledge of lipid species present in the samples. However, the large volume of data generated by the LC–MS analysis made it difficult to process, necessitating a preliminary data compression step. The raw data were compressed while preserving their relevant mass accuracy information to construct more computationally manageable data tables (data matrices). The study utilized the ROIMCR method, (Dalmau et al., 2018), which is based on the combination of the Regions of Interest (ROI) concept (Bedia et al., 2016; Gorrochategui et al., 2015, 2019) and the Multivariate Curve Resolution Alternating Least Squares, MCR-ALS, method (De Juan et al., 2014; Jaumot et al., 2015; Tauler, 1995). The LC elution and mass spectral profiles of the sample constituents (lipids) present in the different examined samples were obtained as a result of the use of this combination approach. ROIMCR is a powerful procedure that has been already described in detail elsewhere (Gorrochategui et al., 2016), and it is summarized in Supporting Information Figs. S1 and S2 and in the text explaining these Figures. ROIMCR can be applied to the analysis of individual chromatographic runs performed on a single sample or to the simultaneous analysis of several related chromatographic runs on multiple samples, including control and TBT treated samples over time. In this second case, the multiple individual data matrices X_i_ generated by the ROI method are vertically concatenated in a column-wise augmented data matrix X_aug_, whose rows are the spectral scans at the retention times of the different chromatographic runs and whose columns are all chromatograms at the different m/z ROI values finally taken into account. For the MCR-ALS simultaneous analysis of the various datasets in this augmented data matrix, neither time alignments nor time shift corrections are required (Gorrochategui et al., 2016, 2019). X_aug_ is decomposed with the MCR-ALS method (De Juan et al., 2014; Jaumot et al., 2015; Tauler, 1995) according to a bilinear factor decomposition model under non-negativity constraints as described in Eq. 1 for I = 1,..n chromatographic runs simultaneously analyzed.

1 $${\text{X}}_{{\text{i}}} {\text{ = C}}_{{\text{i}}} {\text{S}}^{{\text{T}}} {\text{ + E}}_{{\text{i}}}$$

$$\left[ {\begin{array}{*{20}c} {{\text{X}}_{{\text{1}}} } \\ {{\text{X}}_{{\text{2}}} } \\ \ldots \\ {{\text{X}}_{{\text{n}}} } \\ \end{array} } \right]~ = {\text{ X}}_{{{\text{aug}}}} = ~~\left[ {\begin{array}{*{20}c} {{\text{C}}_{{\text{1}}} } \\ {{\text{C}}_{{\text{2}}} } \\ \ldots \\ {{\text{C}}_{{\text{n}}} } \\ \end{array} } \right]{\text{S}}^{{\text{T}}} + \left[ {\begin{array}{*{20}c} {{\text{E}}_{{\text{1}}} } \\ {{\text{E}}_{{\text{2}}} } \\ \ldots \\ {{\text{E}}_{{\text{n}}} } \\ \end{array} } \right] = {\text{ C}}_{{{\text{aug}}}} {\text{S}}^{{\text{T}}} + {\text{E}}$$

In this Equation, the bilinear model gives two factor matrices, C_aug_ and S^T^ In C_aug_ column-wise augmented matrix are the elution profiles of the different components (sample lipid constituents) resolved by MCR-ALS in the different samples, from which the relative concentrations of these components can be obtained using either their peak areas or their peak heights. In the S^T^ matrix are the mass spectra of the resolved components (common to all samples). The resolved components in the mass spectra, can be used for their identification and chemical characterization. This process is commonly known as lipid annotation. The identification and characterization of these resolved components can be performed using public databases such as the Human Metabolome Database (HMBD) (Wishart et al., 2012) as well as the previous laboratory databases developed by our research team.

The study utilized a balanced experimental design to examine the effects of varying levels of TBT dose and time after exposure on the growth of animals. The TBT dose was administered at three levels (C = Control, L = Low, and H = High), while the time after exposure was measured at five levels. Each combination of factor levels was sampled three times to obtain concentration measurements. The factor “time” had five levels since biological samples were collected at 0 h, 8 h, 16 h, 24 h, and 48 h. The factor “dose” had three levels: TBT dose = 0 µg/L (C); TBT dose = 0.1 µg/L (L); and TBT dose = 1 µg/L (H). Thus, the total number of LC–MS chromatographic runs or samples analyzed was 45 (3 × 5 × 3).

The final data matrix included the selected ROI m/z values for all 45 samples analyzed by LC–MS. The study evaluated the effects of TBT exposures over time by examining the peak areas or peak heights of the elution profiles resolved by MCR-ALS for all samples (control and exposed samples) arranged in a new data table (Fig. S1C). The MCR-ALS resolved spectra of the different components were normalized to equal maximum intensity, therefore the concentration profiles of the same component in the different samples change according to their relative concentrations. The peak areas or peak intensities give these relative changes of concentrations of one component (lipid) in the different analyzed samples over treatments and time points.

After the application of the ROIMCR method, the effects of TBT exposure were statistically assessed based on the changes in the peak areas of the resolved elution profiles of the lipidic constituents using different visual and statistical approaches such as Principal Component Analysis (PCA) (Wold et al., 1987), ANOVA (St & Wold, 1989), and ANOVA simultaneous component analysis (ASCA) (Smilde et al., 2005). In addition, a secondary application of the MCR-ALS method was performed to model the changes in the lipidic peak areas of the TBT-treated Daphnia samples over time checking whether these changes can be described by a trilinear factor decomposition model as described in previous work (Malik et al., 2016).

### Visual and statistical analysis of the effects produced by TBT dose and exposure time on the peak areas of the elution profiles resolved by the ROIMCR

The dataset containing the peak areas of all lipid elution profiles was arranged in three data matrices based on the three TBT doses. Each column of peak area matrices represents the concentration changes of each lipid at five different time points. A simple way to graphically represent the effect of the design factors (time and TBT dose) and their interaction on each measured variable (lipid concentration) separately is to plot the lines indicating the trends in the changing values of the variable over time at the different levels of TBT dose (Jafari et al., 2020). This provides an oversimplified, approximative, and qualitative way to measure the effects of the two factors for every lipid data. To evaluate statistically the effects of the two factors, time and TBT dose, and their interaction, a two-way ANOVA method was used, which allowed statistical evaluation of the interaction effects between the two factors. The two-way ANOVA analysis examined the effects and interaction of the two factors on each variable, and these effects were statistically evaluated using an *F*-test. The experimental uncertainty and possible interaction between factors were assessed by using three replicates for each condition.

PCA was used to analyze the overall trend of peak areas of resolved lipid components for different samples, but since it does not take into account the experimental design, its interpretability is limited in cases where datasets are acquired using statistical experimental design.

ANOVA and ASCA models may offer a better view of the overall effects of experimental factors and their interaction on data variation in such cases. ASCA is a statistical analysis technique that combines the benefits of ANOVA and SCA to separate sources of variance and model the individual separate factor effect matrices. In this study, ASCA was used to evaluate the significance of TBT exposure and time by applying a permutation test, where the null hypothesis assumes that the considered factor has no effect. The experimental design used in this work allowed for balanced peak area data matrices to be analyzed, which included the same number of sample replicates for each condition. A more detailed description of the ASCA procedure and permutation tests can be found in previous works (Smilde et al., 2005) and Vis (Vis et al., 2007).

### MCR-ALS bilinear and trilinear modelling of the peak area lipidomics data

The previously described ROIMCR procedure was used to obtain resolved lipids, and their chromatographic peak areas were organized in a data matrix (as mentioned earlier). This matrix can be subjected to further analysis using the MCR-ALS method. In this particular case, the MCR-ALS bilinear model applied to these concentrations data matrix can be written as:

2 $${\text{A}}_{{\text{k}}} = {\text{ T}}_{{\text{k}}} {\text{B}}^{{\text{T}}} + {\text{ E}}_{{\text{k}}} {\text{ k}} = 1{\text{ }}\left( {\text{C}} \right),{\text{ }}2{\text{ }}\left( {\text{L}} \right){\text{ and }}3{\text{ }}\left( {\text{H}} \right)$$

where now the A_k_ is the data matrix with the peak areas (relative concentrations) of the N resolved lipids analyzed over Daphnia samples taken at 5 different times with 3 replicates, under one of the three different doses of TBT, control (C), low (L), and high (H) (k = 1, 2 and 3 for Control, A_C_, low TBT dose, A_L,_ and high TBT dose A_H_) with the same number of rows and columns were (see Supporting Fig. S3).

As shown above, since these three data matrices (C, L, H) have the same dimensions they can be arranged in a data cube, A, or in the column-wise augmented data matrix,

3 $${\text{A}}_{{{\text{aug}}}} = \left[ {{\text{A}}_{{\text{C}}} {\text{;A}}_{{\text{L}}} {\text{;A}}_{{\text{H}}} } \right] = \left[ {\begin{array}{*{20}c} {{\text{A}}_{{\text{C}}} } \\ {{\text{A}}_{{\text{L}}} } \\ {{\text{A}}_{{\text{H}}} } \\ \end{array} } \right] = \left[ {\begin{array}{*{20}c} {{\text{Tc}}} \\ {{\text{T}}_{{\text{L}}} } \\ {{\text{T}}_{{\text{H}}} } \\ \end{array} } \right]{\text{B}}^{{\text{T}}} + \left[ {\begin{array}{*{20}c} {{\text{E}}_{{\text{C}}} } \\ {{\text{E}}_{{\text{L}}} } \\ {{\text{E}}_{{\text{H}}} } \\ \end{array} } \right] = \left[ {{\text{T}}_{{\text{C}}} {\text{;T}}_{{\text{L}}} {\text{;T}}_{{\text{H}}} } \right]{\text{ B}}^{{\text{T}}} + \left[ {{\text{E}}_{{\text{C}}} {\text{;E}}_{{\text{L}}} {\text{;E}}_{{\text{H}}} } \right] = {\text{T}}_{{{\text{aug}}}} {\text{B}}^{{\text{T}}} + {\text{E}}_{{{\text{aug}}}}$$

The resulting three factor matrices T_C,_T_L,_ and T_H_ are related to the time profiles of the different components in the three data matrices C, L, and H, and factor matrix B^T^ gives the lipid composition profiles of the different components obtained by MCR-ALS. E_C,_E_L_and E_H_ give the residual variation not explained by the bilinear model (Eq. 3).

This MCR-ALS bilinear model can be extended to the trilinear model using the application of the trilinearity constraint during the ALS optimization. In matrix form, for every separate data slice (data matrix) the trilinear model equation implies that

4 $${\text{A}}_{{\text{k}}} = {\text{T D}}_{{\text{k}}} {\text{B}}^{{\text{T}}} + {\text{E}}_{{\text{k}}} ~~~~~~~{\text{k}} = 1{\text{ }}\left( {\text{C}} \right),{\text{ }}2{\text{ }}\left( {\text{L}} \right){\text{ and }}3{\text{ }}\left( {\text{H}} \right)$$

Equation 4 describing the trilinear model, agrees with the notation used in MCR bilinear modeling. In the trilinear model, every data matrix at every one of the three treatments (C, L, or H), A_k_, is modeled by the same time profiles, T, and the same lipid composition profiles of the different components, B^T^. The three data matrices only differ in the relative amounts of components given in the three different D_k_ diagonal matrices, k = 1, 2, 3, (C, L, H). The graphical presentation of the MCR-ALS application according to Eqs. 2, 3 and 5 is shown in Supporting Fig. S3.

A previous study (Jafari et al., 2020) showed that the bilinear model (Eqs. 2 and 3) is the best approach to model experimental data when only one experimental factor affects data variability or when two factors have a significant additive effect without interaction. Additional constraints can be applied in the case of application of the bilinear model to reduce the possible rotation ambiguities, but this is out of the scope of this work (Abdollahi & Tauler, 2011; Jaumot & Tauler, 2010). However, the trilinear model (Eq. 4) is more appropriate when a well-defined interaction between two factors produces reproducible multiplicative effects. The trilinear model can directly decompose the three-way dataset into three separate factor matrices, describing variations in TBT dose, time, and lipid composition, without ambiguities. In this work, the best modeling approach for data analysis, either bilinear or trilinear, is investigated after identifying the type of interaction between the design factors in the peak area.

Assessment of the quality of the MCR-ALS models, either bilinear or trilinear, can be performed by calculating the model fitting as model explained data variance (R^2^) or as percentage of lack of fit (lof).

5 $${\text{R}}^{2}=100\times \left(\frac{\sum _{\text{i}=1}^{\text{m}}\sum _{\text{j}=1}^{\text{n}}{\left( {\widehat{\text{x}}}_{\text{ij}} \right)}^{2}}{\sum _{{\text{i}}=1}^{\text{m}}\sum _{{\text{j}}=1}^{\text{n}}{{\text{x}}}_{\text{ij}}^{2}} \right)$$

6 $${\text{lof}}=100\times \sqrt{\frac{\sum _{{\text{i}}=1}^{\text{m}}\sum _{{\text{j}}=1}^{\text{n}}{\left( {\text{x}}_{{\text{ij}}}-{\widehat{\text{x}}}_{\text{ij}}\right)}^{2}}{\sum _{{\text{i}}=1}^{\text{m}}\sum _{{\text{j}}=1}^{\text{n}}{{\text{x}}}_{\text{ij}}^{2}}}$$

where x_ij_ refers to the experimental value in the data matrix and ${\widehat{\text{x}}}_{\text{i}\text{j}}$ is the corresponding calculated value using the MCR-ALS model (either bilinear or trilinear).

## Results and discussion

### Chemometric analysis of LC–MS lipidomics data using the ROIMCR method

The processing of untargeted LC–MS lipidomic experimental data has been improved by using ROI compression, which reduced computer memory requirements by more than 100-fold without any loss of spectral accuracy. The column-wise augmented data matrix was obtained from the 45 individual data matrices corresponding to the analysis of each sample. MCR-ALS was used to extract the elution and spectra profiles of the different individual lipid constituents in the 45 samples analyzed. MCR-ALS initially estimates the number of components that explain the variance of the data matrix, typically using the singular value decomposition (SVD) method. This estimation should encompass the lipids present in the sample, as well as other possible contributions from background, solvent, and other instrument artifacts. The final selection of the number of components is a compromise that considers the explained data variance on one side and the incorporation of all possible significant chemical sources of data variance on the other side. Therefore, adding additional components will not provide the resolution of extra components with reasonable shapes of their elution and spectral profiles.

Ultimately, 87 of the components resolved by MCR-ALS could be assigned to lipid constituents, characterized, and identified, still explaining 96.05% of the experimental data variance. The initial estimates of the S^T^ matrix are derived from the more distinct rows (MS spectra) of the data matrix (see Winding & Guilment, 1991). The elution profiles of these components in the different samples and of their mass spectra were properly resolved using the MCR-ALS method under only non-negativity constraints and spectra normalization. The untargeted analysis used in this work provided an additional number of lipids (19 lipids) showing statistically significant changes in their concentration due to TBT exposure and time, as given in Supporting Table 1S. The advanced chemometrics data analysis methods like MCR-ALS are necessary to gain a deeper understanding of the experimental lipidomics data obtained in this work because the LC–MS chromatograms exhibited complex profiles with multiple coeluted compounds, making it difficult to detect and identify directly what lipids changed their concentration over time and at the different TBT doses.

**Fig. 1:**
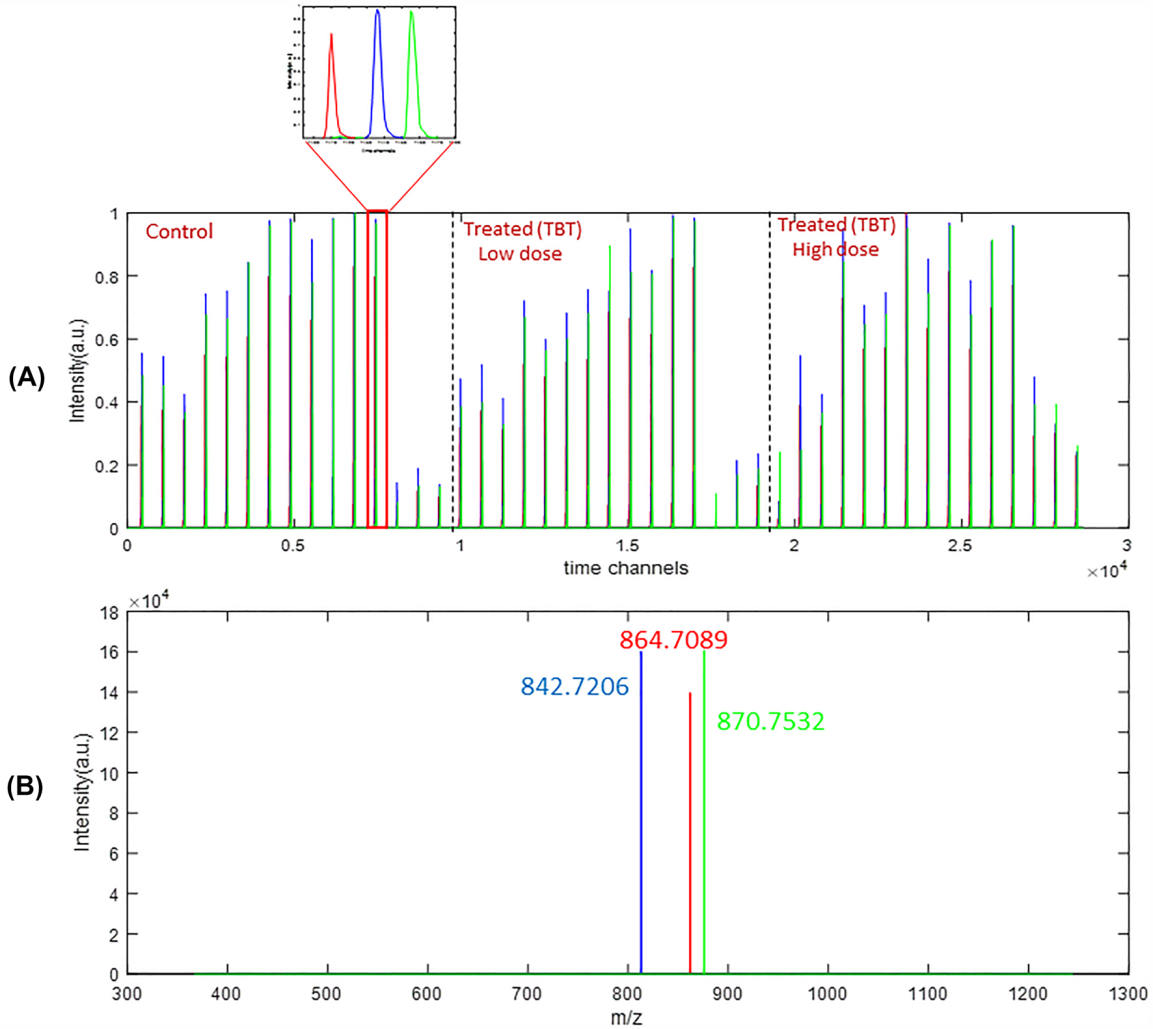
**A** MCR-ALS resolution of the elution profiles of three components (lipids) in 45 samples. In the zoomed view, the MCR-ALS resolved elution profiles for these three lipids is shown in more detail for a control sample. **B** MCR-ALS resolution of the mass spectra of these three lipids, which were identified as TAG 50:5 (Blue), TAG 52:8 (red), TAG 52:5 (green)

Using the elution profiles of the same component in the different samples, it was then possible to analyze the variation in peak areas between them. As an example, Fig. 1 displays the MCR-ALS resolution of the pure elution and spectral profiles of a reduced group of three coeluted lipids in the 45 samples analyzed (X_aug_ matrix). The zoomed view of the elution profiles of one of the control samples shows three different lipids. The pure mass spectra of these three lipids are also shown in the lower part of Fig. 1. These spectra were identified as the lipids TAG 50:5, TAG 52:8, TAG 52:5 as given in Supporting Table 1.

**Fig. 2:**
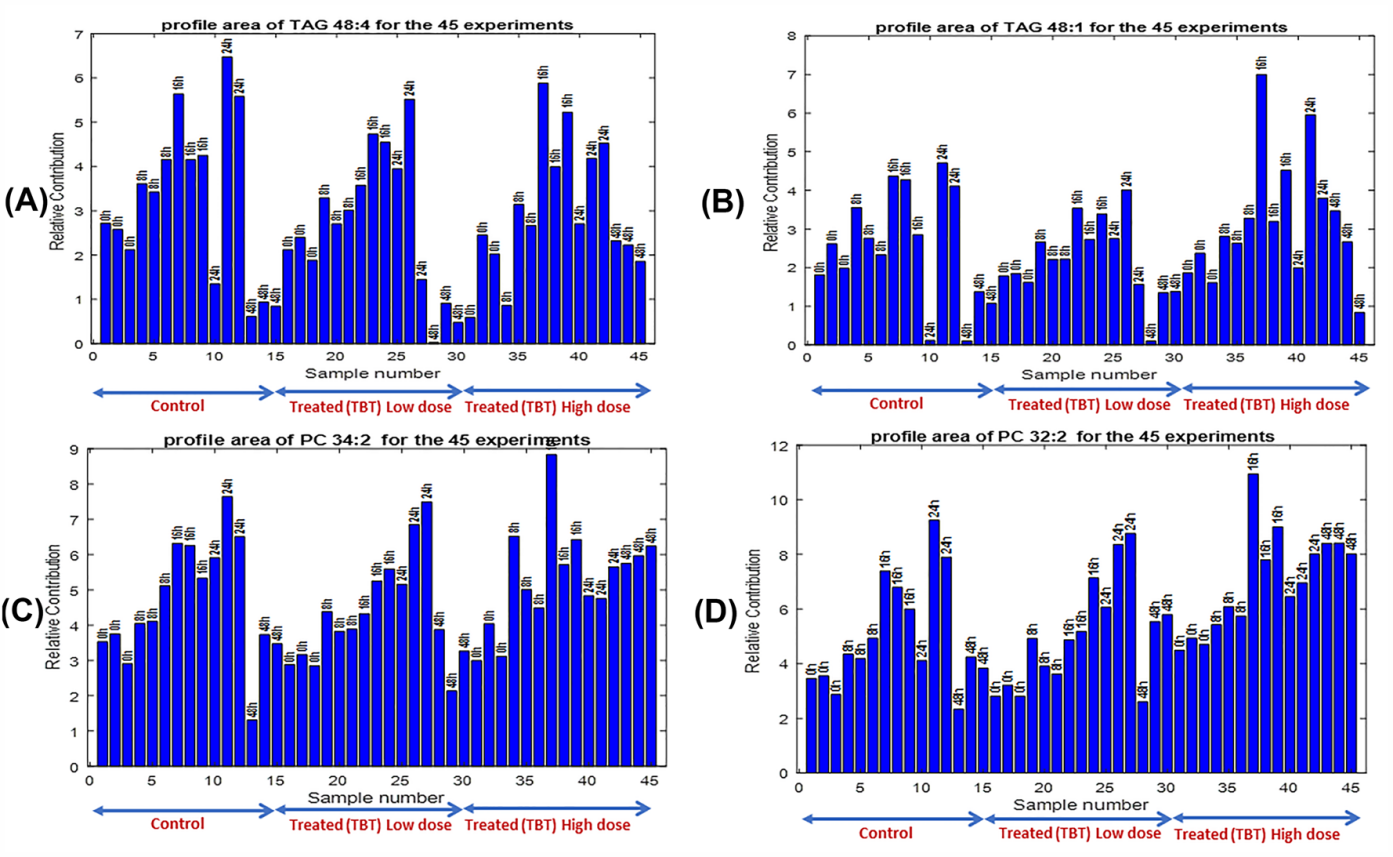
Peak areas of the MCR-ALS resolved elution profiles of four lipids in the 45 samples simultaneously analyzed (control, TBT low dose, and TBT high dose). **A** TAG 48:4, **B** TAG 48:1, **C** PC 34:2 and **D** PC 32:2

In Fig. 2, the peak areas of the four lipids (TAG 48:4, TAG 48:1, PC 34:2 and PC 32:2) (see Supporting Table 1) in the forty-five samples simultaneously analyzed are given. In the four cases an increase of concentrations (peak areas) of these lipids is first observed over time, with a sudden decrease at the last times. In every one of the four lipid plots, there are three peak areas groups which correspond to control, low and high TBT level treated samples. It is still difficult to see from this first representation of the results for these four lipids if there is a significative effect due to TBT dose. The significance of the two factors, time and TBT dose level, and of their interaction are better investigated in the following data analysis results.

#### Univariate graphical visualization of the effects of the factors and their interaction

A visual exploratory analysis of the effects of design factors (time and TBT dose) and their possible interactions on individual response variables (lipid concentration) was initially carried out using so-called interaction plots. All variables (87 lipid concentrations) were analyzed individually using this method and examined for the effect of the two factors and their possible interaction. Due to experimental noise in real data, it was not easy to interpret these interaction plots. The effects of design factors, time and TBT dose, on individual response variables, specifically lipid concentration, were explored through interaction plots. However, due to experimental noise, it is challenging to interpret the plots, and it is difficult to extract clear conclusions about the type of effect due to the dose treatment. An example of the visualization is provided in Fig. S5, showing the changes in peak areas of four lipids, as well as their concentration over time and at three TBT doses in control, low, and high samples. While some lipids seem to have a dependency on the dose level, the lines are not parallel, making it difficult to draw any definitive conclusions.

#### PCA

The application of PCA to the data matrix of the autoscaled ROIMCR resolved elution profiles peak areas allowed the exploration of the main data patterns. Four PCs explain over 80% of the variance, with the first two already explaining 73.53%. In the PCA scores plot, samples exposed to TBT for 24 h have higher positive scores, while those exposed for 48 h have negative scores. The loading plots show that unsaturated TAGs and PC have high positive loadings, with moderate loadings on unsaturated DAGs and PE in PC1. PC2 shows higher positive scores in a few 48 h samples with a high TBT dose. The lipid composition of the samples changed at 24 h, with a further deviation at 48 h with a high TBT dose, as levels of unsaturated TGs and PC increased and then decreased after allocation to molt and eggs.

#### Two-way ANOVA evaluation of the significance of the factors and their interaction

Table 1 summarizes the results of the ANOVA evaluation of the effects of time and TBT dose on individual lipid responses. The influence of the two factors and their interaction varied for each lipid. The concentration of the TAG 48:4 changed only under the influence of the time factor, whereas the levels of the PC 34:2 and PC 32:2 were affected by both factors and their interaction. Time significantly affected the concentration of 78 out of 87 lipids, while 30 lipids had their levels altered by the TBT dose factor, and 14 were affected by the interaction between the two factors. The amount of data variance of each variable explained by the factors can be deduced from the sum of squares and F ratios in ANOVA Table 1, and the significance of these contributions can be evaluated statistically. Time and TBT dose factors were significant, and an interaction between the two was also significant (P < 0.01), but the types of TBT dose effects cannot be determined.

**Table 1:** Two-way ANOVA results

Effects	TAG 48:4	TAG 48:1	PC 34:2	PC 32:2
Dose SS^a^	2.066	27.630	8.251	39.88
Time SS	72.09	38.180	56.21	90.41
Inter. SS	8.923	40.256	23.48	27.72
Error SS	35.41	35.413	12.02	29.03
Dose F^b^	0.88	2.86	5.87	15.33
Time F	15.27	7.15	19.9	17.38
Interact F	0.95	0.36	4.02	2.66
P^c^ Dose > F	0.4271	**< 0.01**	**< 0.01**	**< 0.01**
P Time > F	**< 0.01**	**< 0.01**	**< 0.01**	**< 0.01**
P Inter. > F	0.4952	0.9333	**< 0.01**	**< 0.01**

Bold numbers: Significance

^a^The sum of squares due to each source

^b^F-statistic, which is the ratio of the mean squares

^c^The p-value, which is the probability that the F-statistic can take a value larger than the computed test-statistic value

#### Multivariate analysis of evaluation of the significance of the factors and their interaction using variance-simultaneous component analysis (ASCA)

In addition to the previous univariate analysis of the effects of the design factors and their interaction on each lipid concentration separately using the two-way ANOVA, the same effects were evaluated simultaneously for all lipids concentrations by the ASCA method, as a multivariate extension of ANOVA.

Table 2 presents the results of applying ASCA to the 87 variables (lipid peak areas) with statistically significant effects for the dose, time, and time × dose interaction sub-models. The time factor had the most significant influence on lipid profiles, covering about 62% of the total variation. The contribution of the dose factor was 7%, and the interaction factor was 21%. The residual variance was 9%, representing the variance among replicates. The interaction sub-model was statistically significant, suggesting that the time evolution of lipid concentrations was dependent on the applied TBT dose. The permutation test validated the ASCA model, providing statistically significant effects for all sub-models with p-values ranging from 0.0001 to 0.0038.

**Table 2:** ASCA results

	Effect	Prob > F
Dose	6.83	**0.0001**
Time	62.25	**0.0034**
Inter.	21.69	**0.0038**
Error	9.23	

Bold numbers: Significance

**Fig. 3:**
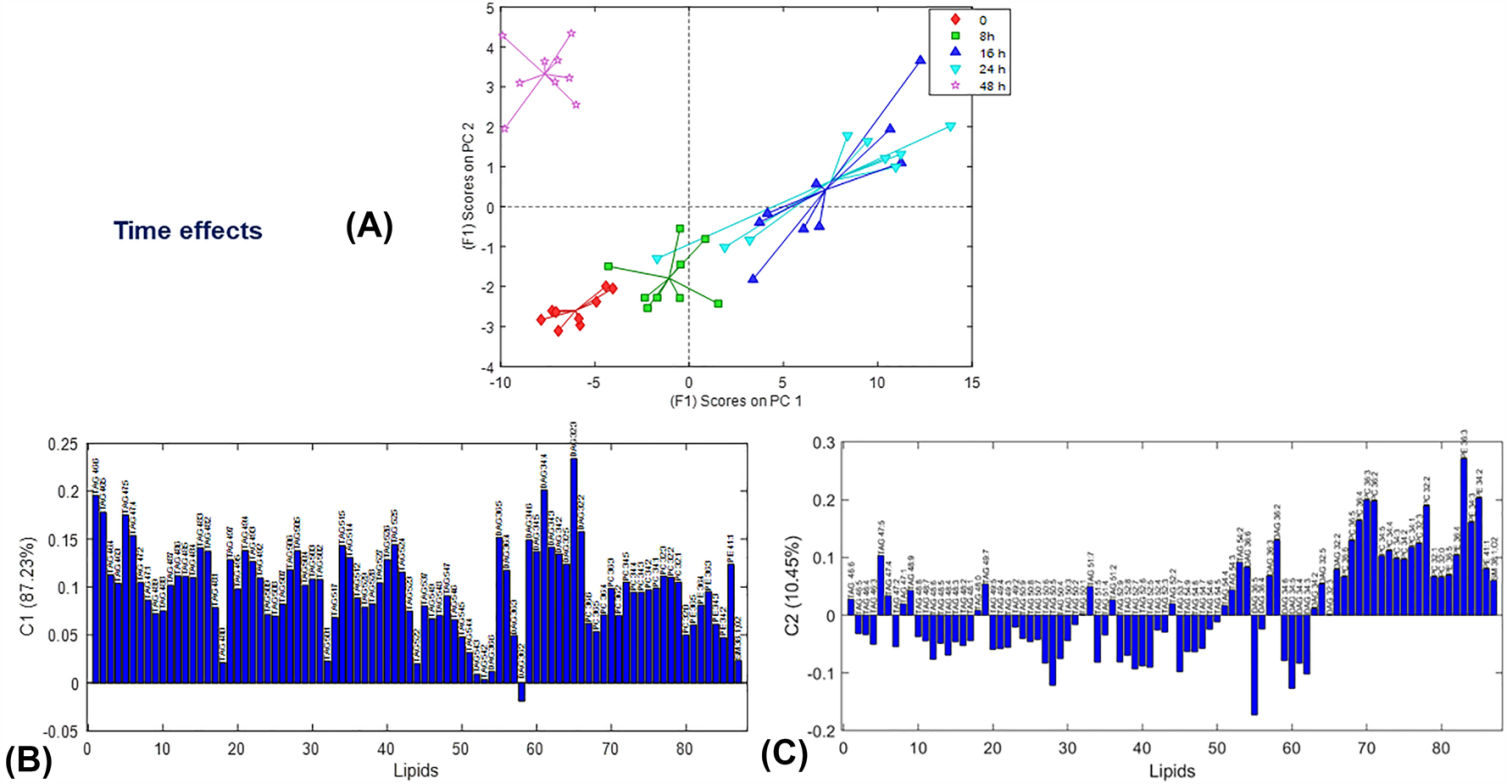
Time effects on lipids by ASCA model in the analysis of the Table of the peak areas of the MCR-ALS resolved elution profiles. **A** ASCA scores plot and **B** ASCA loadings for the first component (C1), and **C** ASCA loadings for the second component (C2)

Figure 3A–C presents the scores and loadings plots for the time factor ASCA sub model, which showed that the first component (C1) explained 87% of the variance, indicating gradual changes in the lipid concentrations over time, with a sudden drop in levels at 48 h (after molting and producing the first clutch of eggs). This pattern can be related to the storage of lipids acquired from food during the intermolt period (8–24 h). In C2, sample scores increased after 0 h, reaching their highest levels at 48 h, with positive loadings for abundant phospholipids PC 34:1–5, PC 36:2–6, most PEs, and C16SM.

**Fig. 4:**
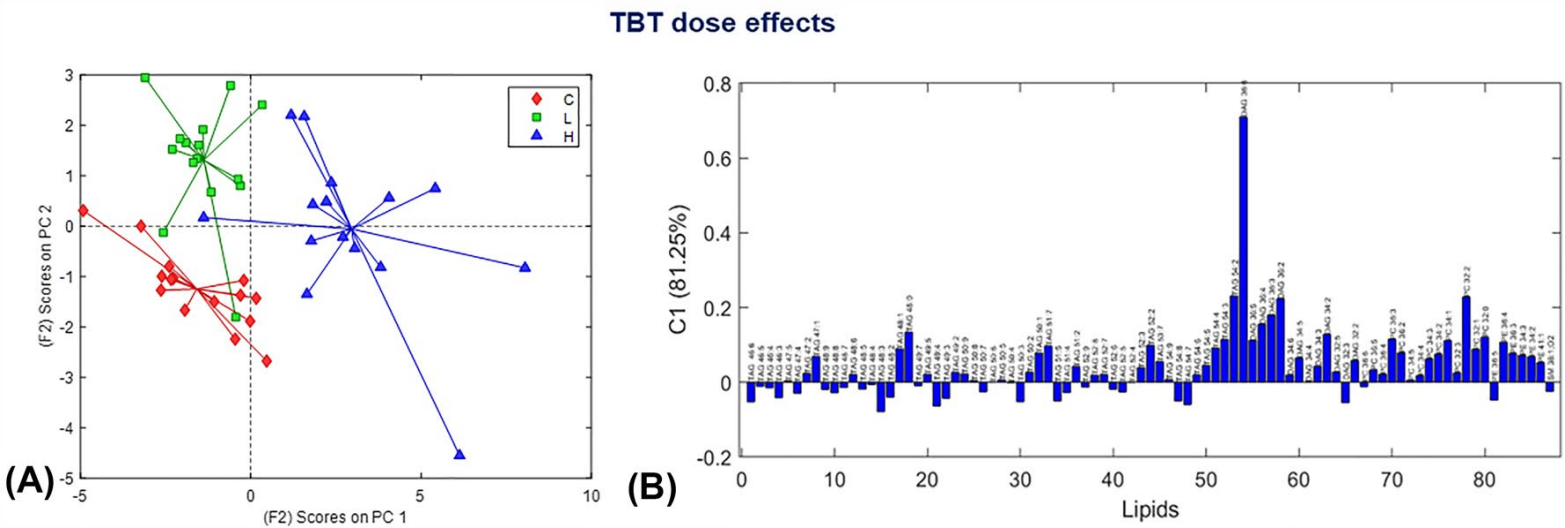
Dose effects on lipids by ASCA model in the analysis of the Table of the peak areas of the MCR-ALS resolved elution profiles. **A** ASCA scores plot, and **B** ASCA loadings plot for the first component (C1)

**Fig. 5:**
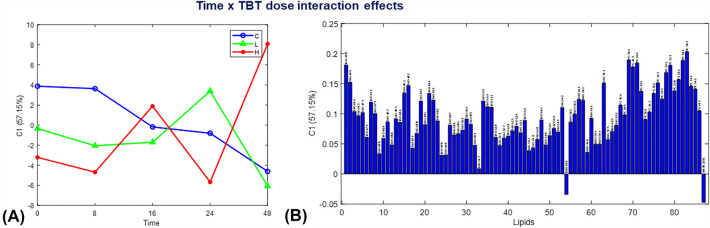
Interaction ‘dose × time’ effects on lipids by ASCA model in the analysis of the Table of the peak areas of the MCR-ALS resolved elution profiles. **A** ASCA scores, and **B** ASCA Loadings for the first component (C1)

The effect of TBT was studied at three levels, including control non-treated TBT samples group, and low-level and high-level TBT dose treated samples. The maximum number of components in the ASCA model that could be fitted was two. The ASCA model computed on the ‘dose’ effect matrix already explained about 81% of the variance using one first component (C1) only, and the second component captured the remaining variance (29%). The ASCA scores plot for the ‘dose‘ effect is presented in Fig. 4A. Along the C1 axis, the samples were grouped according to their three levels of TBT dose; with the samples treated with high TBT concentration ’H‘ appearing with positive scores and the control samples ‘C’ (no TBT added) and low TBT concentration ‘L’ appearing mostly with negative scores. The loadings plot for C1 (Fig. 4B) shows that the TBT dose increased the levels of polyunsaturated members of the most abundant TAGs (TAG 50:1, TAG 51:7, TAG 52:2, TAG 54:2–5), and DAGs (DAG 36:2–4), and PC (PC 32:0, PC 32:2), and of the majority of PE (PE 34:2, PE 36:4). These results confirm previous findings (Jordão et al., 2015) that indicate that TBT impaired the allocation of specific lipids to eggs, which remained in de-brooded females Fig. 5.

The ASCA scores profiles of the interaction sub model show how the time and TBT dose factors interact in *Daphnia magna* samples (Figure 5). The scores profile of the first component, explaining over 57% of data variance, indicates a more significant effect of high TBT dose on lipid profiles at 48 h compared to control and low TBT dose treatments. High TBT doses decreased lipid accumulation at the beginning of the instar period and produced higher levels of lipids at 48 h, which is different from the pattern observed for control and low TBT dose treatments. The results obtained by ASCA are similar to those of a previous targeted analysis study, and the MCR-ALS method can complement the findings.

#### Bilinear and trilinear MCR-ALS modelling of the datasets

The complexity of the three-way dataset built from the peak areas of the MCR-ALS resolved elution profile at the different experimental conditions was first evaluated using SVD analysis of the column- and row-wise augmented data matrices (see Supporting Fig. S6). Four larger similar size singular values were obtained in both cases, describing approximately the same amount of data variance. According to the results obtained in previous work (Jafari et al., 2020), column- and row-wise augmentation datasets give the same number of major components when the factor interaction effects are multiplicative. Therefore, this will be in agreement with a trilinear model data structure and with the presence of multiplicative effects between the two design factors, TBT dose and time after exposure.

**Fig. 6:**
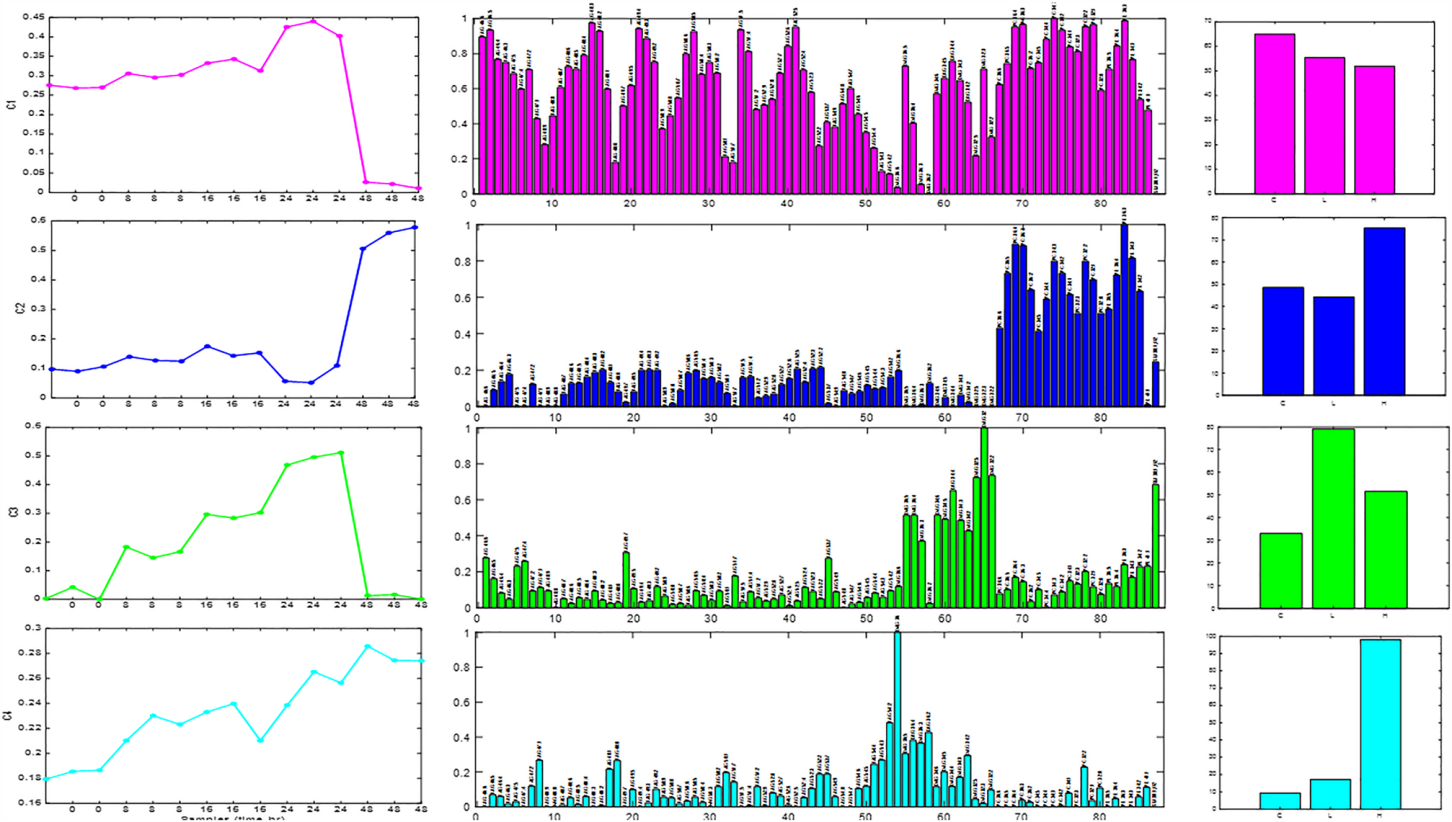
MCR-ALS resolved profiles in the samples (time), lipids, and dose modes of the three-way dataset

The trilinear model constraint in MCR-ALS (Tauler, 2021; Tauler et al., 2020) was applied to the data set, and it did not result in poorer fitting results than the bilinear model. The initial estimates for the S^T^ matrix were derived from the more distinctive rows of the MS data matrix (Winding & Guilment, 1991). During the MCR-ALS analysis, two constraints were applied, spectra and elution profiles non-negativity, and spectral normalization (equal height). The trilinear model constraint eliminated the rotation ambiguity involved in the bilinear factor decomposition of the same data set. Using a four-component MCR-ALS bilinear model, the amount of explained data variance was 95.05%, whereas with the same number of components using the trilinear model constraint, the explained variance was 93.95%. The profiles resolved by either bilinear or trilinear models were rather similar, and the analyzed experimental data behaved in a rather good trilinear way. The MCR-ALS profiles of the four components in the three data modes are shown in Fig. 6 and have been ordered according to their explained variances.

The MCR-ALS analysis of the lipid profiles identified four components that explain 23–43% of the variance in the data. Component 1 showed a decrease in the lipid effects of TBT at low and high doses, while component 2 showed an increasing effect of TBT at high dose levels. Component 3 showed a steady increase in sample loadings over time, with a drop after 48 h, and the effects of the lower TBT dose were more significant. Component 4 showed a significant increase in the concentrations of unsaturated TAGs and DAGs, with the highest levels at 24–48 h, induced by the highest TBT dose treatment (Grün & Blumberg, 2006), consistent with the obesogenic effect of TBT. The degree of unsaturation increased with the length of the carbon chain in TAGs and DAGs, similar to the lipid profile reported for *Daphnia magna* food (Fuertes et al., 2018).

In summary the C1 profile is loaded mostly by polyunsaturated TAGs and phosphocholine (PC), the C2 profile is mostly loaded by phosphocholine (PC), phosphatidylethanolamine (PE) and C3 by those DAGs that decreased dramatically over 48 h, which were then accumulating to a greater extent when exposed to low levels of TBT. The direct precursors of TAGs are DAGs were formed from phosphatidic acid produced either by the glycerophosphate pathway, by the monoacylglycerol pathway, or by the degradation of phospholipids or catabolism of TAGs (Arrese & Soulages, 2010). In the C3 profiles, most lipid classes had moderate loadings. It can be concluded that TBT disrupts the synthesis of TAGs from DAGs in Daphnia exposed to TBT which showed increased levels of lipid droplets during the entire molt cycle (Jordão et al., 2015). Finally, C4 profiles separated Daphnia exposed to TBT at 24 and 48 h from those not exposed. and they had higher loadings for saturated or unsaturated TAGs and DAGs. This means that Daphnia exposed to TBT accumulated less unsaturated TAGs and DAGs. These results agreed with the results obtained in previous works (Malik et al., 2016).

## Conclusions

In this study, we employed a non-targeted lipidomics ROIMCR approach to identify a substantial number (87) of lipids in *Daphnia magna* that serve as reliable indicators of the effects resulting from exposure to TBT dose, as well as the time after of exposure. Multivariate data analysis techniques, including ASCA and MCR-ALS, proved to be particularly valuable in extracting meaningful biological information from the datasets analyzed, allowing us to discern changes in the concentrations of various lipid profiles in *Daphnia magna* following the dose of exposure to TBT, as well as the duration of exposure.

A comparison of the results obtained from this non-targeted study analysis with those of a previous targeted analysis of the same samples revealed that, in this non-targeted study, a greater number of lipids were resolved, and statistically significant changes in their concentrations due to TBT exposure and time were detected. The use of current lipidomics databases enabled the identification of 87 lipids, 19 of which were newly identified in this work. However, 18 lipids still remain unidentified. Notably, TAG 46:6, TAG 46:5, TAG 46:4, TAG 46:3, TAG 47:5, TAG 47:4, TAG 47:2, TAG 47:1, TAG49:7, TAG 49:5, TAG 49:4, TAG 49:3, TAG 49:2, TAG 51:7, TAG 51:5, TAG 51:4, TAG 51:2, TAG 53:7, and PC 32:3 are among the newly identified lipids.

The findings of this study validate the conclusions drawn in our previous work, which utilized various synthetic data sets and examined different types of factor interactions (Jafari et al., 2020). Our analysis of real experimental lipidomics data indicates that the interaction between the two factors considered in this study (TBT dose and time after exposure) was multiplicative in nature. Moreover, the most suitable model for analyzing this type of three-way lipidomics data was found to be the trilinear model, which produced accurate and reliable results. Therefore, these results confirm that the trilinear factor decomposition model is appropriate for cases involving reproducible multiplicative effects between design factors, and that the use of MCR-ALS with non-negativity and trilinearity constraints is essential for optimal recovery of omics profiles.

## Supplementary Information

Below is the link to the electronic supplementary material.

Supplementary material 1 (DOCX 1511.9 kb)
