# Supplementary material for: Non-target ROIMCR LC–MS analysis of the disruptive effects of TBT over time on the lipidomics of Daphnia magna
Source: Metabolomics. 2023 Aug 7;19(8):70. doi: 10.1007/s11306-023-02030-w (PMC10406683; doi:10.1007/s11306-023-02030-w)
Supplement: Supplementary file 1 — Supplementary material 1 (DOCX 1511.9 kb) [file 11306_2023_2030_MOESM1_ESM.docx]

**Supporting Information**

**Non-target ROIMCR LC-MS analysis of the disruptive effects of TBT over time on the lipidomics of *Daphnia magna***

Jamile Mohammad Jafari^a^, Josefina Casas^b,c^, Carlos Barata^d^, Hamid Abdollahi^a^, Romà Tauler^d^*

^a^ Institute for Advanced Studies in Basic Sciences (IASBS), Zanjan, F: (+98) 24 3315 3232, T:(+98) 24 3315 3122. Iran

^b^ RUBAM, Institute for Advanced Chemistry (IQAC-CSIC), Jordi Girona 18-26, Barcelona 08034, Spain

^c^ Liver and Digestive Diseases Networking Biomedical Research Center (CIBEREHD), Instituto de Salud Carlos III, 28029 Madrid, Spain

^d^ Department of Environmental Chemistry, Institute of Environmental Assessment and Water Research (IDAEA), Spanish Research Council (IDAEA-CSIC), Barcelona, Spain, F:(+34) 93 204 59 04, T(+34) 93 400 61 40,

[*Roma.Tauler@idaea.csic.es](mailto:*Roma.Tauler@idaea.csic.es)


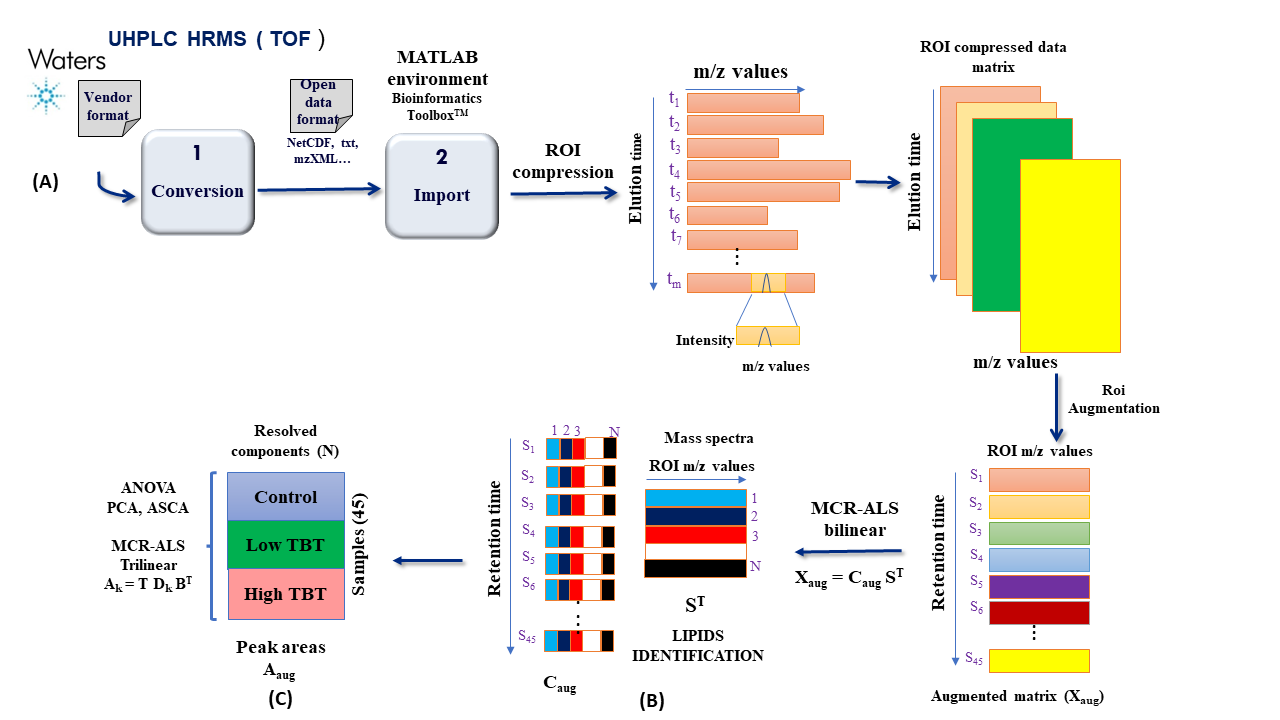


**Figure S1.** Workflow of the ROIMCR data analysis method (Gorrochategui *et al*, 2019). Matrix **X_aug_** is decomposed into two factor matrices: **C_aug_** which has the resolved elution profiles of the N components in the different simultaneously analyzed samples (chromatographic runs), and **S^T^**, which has the mass spectra of these N resolved components.

Raw data files from the LC-MS analysis of each sample were converted to CDF format using the Databridge function of MassLynx™ V. 4.1 software. LC-MS Data were uploaded to the MATLAB Workspace environment (version R2018b, The Mathworks Inc.). LC- MS data were arranged in a data matrix, with retention times in the rows and selected m/z values in the columns. The selection of the Regions Of Interests or ROIs, allows for the selection of m/z traces whose intensity signals are higher than a preselected threshold value (above instrumental noise). This approach gives chromatographic MS matrices with the highest experimental mass accuracy with low memory requirements. Different data matrices were created for each sample analyzed (Figure S1A) which were further processed and vertically concatenated into a large column-wise augmented data matrix (**X_aug_**) which was then analyzed by the MCR-ALS (De Juan *et al*, 2014; Jaumot *et al*, 2015; Tauler, 1995) method.


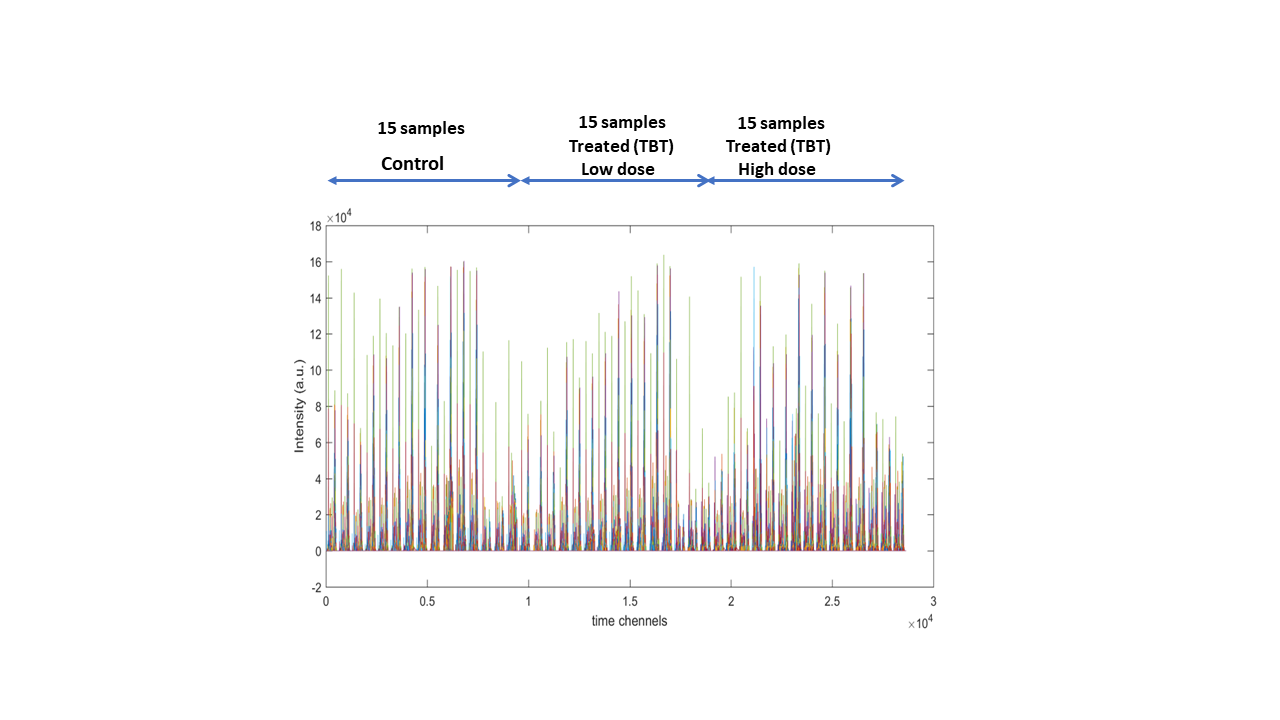
**Figure S2.** LC-MS ROI chromatograms of 45 samples under the three doses of TBT (Control, Low, High). and over five times (0, 8, 16, 24, 48), give the **X_aug_** column-wise augmented data matrix. On the left of the plot, the 15 control samples, on the middle of the plot, the 15 Low dose of TBT samples, and on the right of the plot, the 15 High dose of TBT samples.

Figure S2 displays LC-MS chromatograms after ROI compression (**X_aug_**). MCR-ALS decomposes the experimental data sets extended to the simultaneous analysis of a large number of chromatographic runs. For example, in the case of this work, each of the column-wise augmented data matrices (**X_aug_**) had the information related to the 45 single LC-MS data matrices. **X_aug_** matrices were decomposed by MCR-ALS method as shown in Equation 2 and Figure S1B.


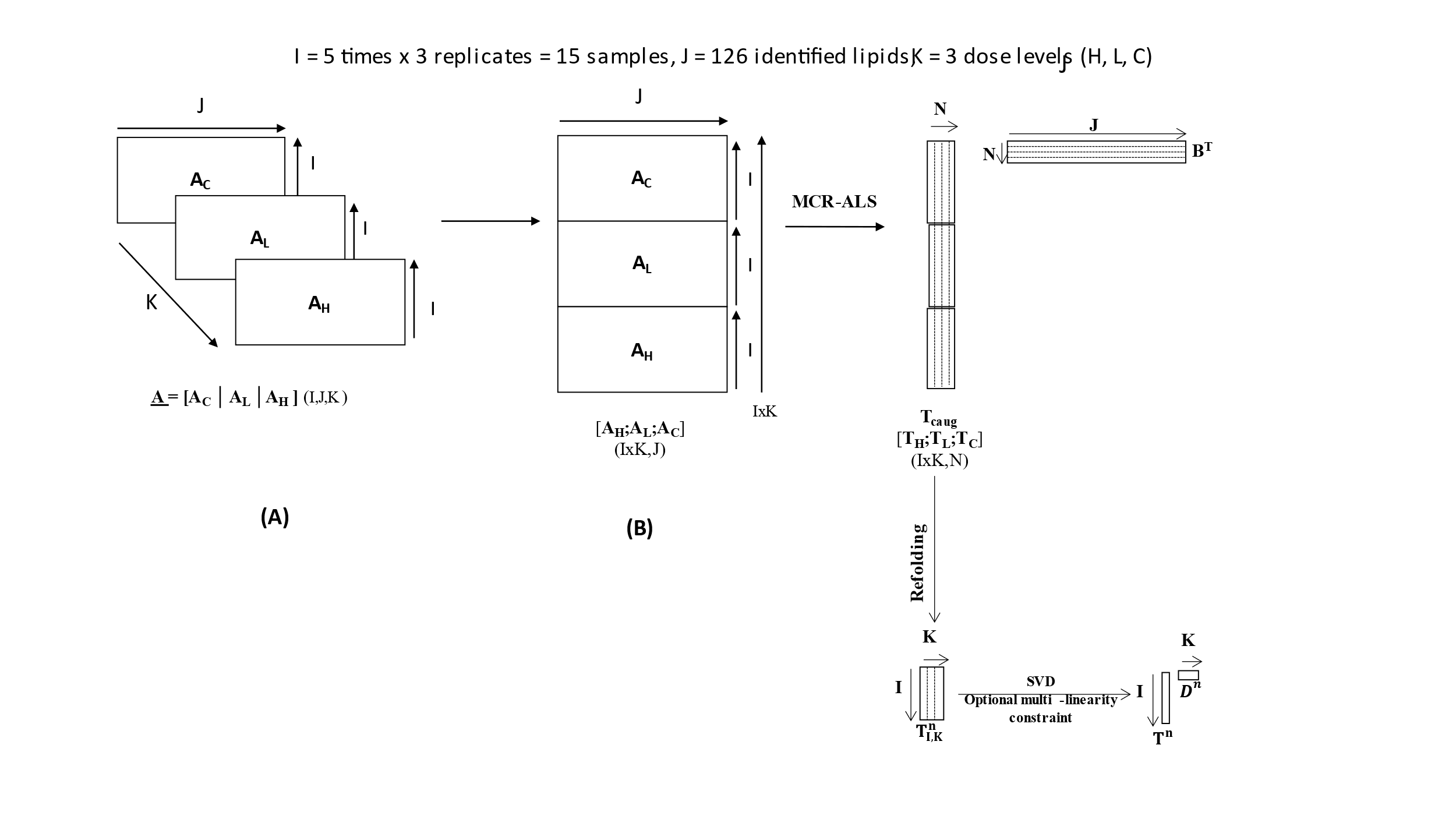


**Figure S3.** Graphical representation of the MCR-ALS analysis of the peak areas data matrices **A_C_, A_L_, A_H_** : (A) Three-way data set (data cube) arrangement **A** of dimensions (15,126,3), (B) column-wise matrix augmentation data arrangement, **A_aug_ = [A_C_;A_L_;A_H_]** and resolution of the profiles in the three datra modes (T: samples/time mode; B: variables mode (lipids); and D: treatment dose mode).

**Supporting Table S1. Identification of the 87 lipids resolved by MCR-ALS**

| **Identified Lipids** | **Adduct*** | **m/z** | **Retention time** |
| --- | --- | --- | --- |
| TAG 46:6 | M+NH4+H | 785.6471 | 12.8521 |
| TAG 46:5 | M+NH4+H | 787.6650 | 13.4740 |
| TAG 46:4 | M+NH4 | 788.6714 | 14.0657 |
| TAG 46:3 | M+NH4 | 790.6880 | 14.6572 |
| TAG 47:5 | M+NH4+H | 801.6803 | 13.9085 |
| TAG 47:4 | M+NH4+H | 803.6971 | 14.4729 |
| TAG 47:2 | M+NH4 | 806.7202 | 15.5272 |
| TAG 47:1 | M+NH4 | 808.7350 | 16.0953 |
| TAG 48:9 | M+NH4 | 806.6300 | 11.8571 |
| TAG 48:8 | M+NH4  M+NH4+H | 808.6429  809.6485 | 12.5411 |
| TAG 48:7 | M+NH4  M+NH4+H | 810.6576  811.6646 | 13.1631 |
| TAG 48:6 | M+NH4 | 812.6727 | 13.7850 |
| TAG 48:5 | M+NH4  M+NH4+H | 814.6877  815.6972 | 14.3768 |
| TAG 48:4 | M+NH4 | 816.7048 | 14.9057 |
| TAG 48:3 | M+NH4 | 818.7214 | 15.4347 |
| TAG 48:2 | M+NH4 | 820.7407 | 15.9026 |
| TAG 48:1 | M+NH4 | 822.7524 | 16.3638 |
| TAG 48:0 | M+NH4 | 824.7680 | 16.8934 |
| TAG 49:7 | M+NH4 | 824.6752 | 13.6016 |
| TAG 49:5 | M+NH4 | 828.7061 | 14.6595 |
| TAG 49:4 | M+NH4 | 830.7212 | 15.1858 |
| TAG 49:3 | M+NH4 | 832.7384 | 15.6529 |
| TAG 49:2 | M+NH4  M+NH4+H | 834.7527  835.7584 | 16.1483 |
| TAG 50:9 | M+NH4  M+NH4+H | 834.6685  835.6707 | 12.7904 |
| TAG 50:8 | M+NH4 | 836.6753 | 13.4128 |
| TAG 50:7 | M+NH4  M+NH4+H | 838.6902  839.6953 | 13.9704 |
| TAG 50:6 | M+NH4  M+NH4+H | 840.7065  841.7092 | 14.5334 |
| TAG 50:5 | M+NH4  M+NH4+H | 842.7206  843.7296 | 15.0631 |
| TAG 50:4 | M+NH4  M+NH4+H | 844.7381  845.7396 | 15.5918 |
| TAG 50:3 | M+NH4  M+NH4+H | 846.7534  847.7599 | 16.0564 |
| TAG 50:2 | M+NH4 | 848.7685 | 16.5323 |
| TAG 50:1 | M+NH4  M+NH4+H  M+NH4+(2*H) | 850.7842  851.7885  852.7968 | 17.0898 |
| TAG 51:7 | M+NH4 | 852.7100 | 14.3461 |
| TAG 51:5 | M+NH4 | 856.7348 | 15.3424 |
| TAG 51:4 | M+NH4 | 858.7535 | 15.9044 |
| TAG 51:2 | M+NH4 | 862.7831 | 16.8627 |
| TAG 52:9 | M+NH4 | 862.6934 | 13.5976 |
| TAG 52:8 | M+NH4 | 864.7089 | 14.1888 |
| TAG 52:7 | M+NH4  M+NH4+H | 866.7199  867.7292 | 14.7207 |
| TAG 52:6 | M+NH4  M+NH4+H | 868.7369  869.7431 | 15.2165 |
| TAG 52:5 | M+NH4  M+NH4+H | 870.7532  871.7579 | 15.7148 |
| TAG 52:4 | M+NH4  M+NH4+H | 872.7680  873.7726 | 16.2155 |
| TAG 52:3 | M+NH4  M+NH4+H | 874.7832  875.7921 | 16.7172 |
| TAG 52:2 | M+NH4  M+NH4+(2*H) | 876.7998  878.8148 | 17.2785 |
| TAG 53:7 | M+NH4 | 880.7436 | 15.0335 |
| TAG 54:9 | M+NH4  M+NH4+H | 890.7259  891.7271 | 14.3490 |
| TAG 54:8 | M+NH4 | 892.7371 | 14.8750 |
| TAG 54:7 | M+NH4  M+NH4+H | 894.7508  895.7562 | 15.4037 |
| TAG 54:6 | M+NH4  M+NH4+H | 896.7689  897.7748 | 15.9108 |
| TAG 54:5 | M+NH4  M+NH4+H | 898.7846  899.7883 | 16.4058 |
| TAG 54:4 | M+NH4 | 900.8002 | 16.9359 |
| TAG 54:3 | M+NH4 | 902.8152 | 17.4971 |
| TAG 54:2 | M+NH4  M+NH4+H  M+NH4+(2*H) | 904.8309  905.8370  906.8450 | 18.1190 |
| DAG 36:6 | M+NH4 | 630.4964 | 5.083 |
| DAG 36:5 | M+NH4 | 632.5237 | 7.9381 |
| DAG 36:4 | M+NH4 | 634.5391 | 8.8466 |
| DAG 36:3 | M+NH4 | 636.5540 | 9.7486 |
| DAG 36:2 | M+NH4 | 638.5703 | 10.6518 |
| DAG 34:6 | M+NH4 | 602.4768 | 5.9785 |
| DAG 34:5 | M+NH4 | 604.4920 | 6.6961 |
| DAG 34:4 | M+NH4 | 606.5083 | 7.5652 |
| DAG 34:3 | M+NH4 | 608.524 | 8.4987 |
| DAG 34:2 | M+NH4 | 610.5385 | 9.3711 |
| DAG 32:5 | M+NH4 | 576.4613 | 5.6399 |
| DAG 32:3 | M+NH4 | 580.4929 | 7.1956 |
| DAG 32:2 | M+NH4 | 582.5083 | 8.0651 |
| PC 36:6 | M+H | 778.5389 | 5.8836 |
| PC 36:5 | M+H  M+(2*H) | 780.5531  781.5584 | 6.5737 |
| PC 36:4 | M+H  M+(2*H) | 782.5685  783.5729 | 7.4392 |
| PC 36:3 | M+H | 784.5823 | 8.3723 |
| PC 36:2 | M+H  M+(2*H) | 786.5972  787.6013 | 9.3696 |
| PC 34:5 | M+H | 752.5218 | 5.6397 |
| PC 34:4 | M+H  M+(2*H) | 754.5375  755.5415 | 6.2931 |
| PC 34:3 | M+H  2*(M+H)  2*(M+H)+(2*H)  M+(2*H) | 756.5537  1512.101  1514.111  757.5595 | 7.1289 |
| PC 34:2 | M+H  M+(2*H)  2*(M+H) | 758.5683  759.5714  1516.127 | 7.9684 |
| PC 34:1 | M+H  M+(3*H) | 760.5826  762.5958 | 8.9014 |
| PC 32:3 | M+H | 728.5231 | 6.0744 |
| PC 32:2 | M+H  M+(2*H) | 730.5399  731.5445 | 6.7253 |
| PC 32:1 | M+H | 732.5529 | 7.6575 |
| PC 32:0 | M+H  M+(2*H) | 734.5669  735.5719 | 8.4982 |
| PE 36:5 | M+H | 770.5699 | 7.7191 |
| PE 36:4 | M+H  M+H | 740.5216  741.5266 | 7.4392 |
| PE 36:3 | M+H | 742.5365 | 8.3723 |
| PE 34:3 | M+H | 714.5057 | 7.1289 |
| PE 34:2 | M+H  M+(2*H) | 716.5221  717.5387 | 7.9993 |
| PE 41:1 | M+H-H_2_O | 798.6593 | 13.3174 |
| SM 38:1;O2 | M+H | 759.6322 | 10.2449 |

|  |  |  |  |  |  |  |
| --- | --- | --- | --- | --- | --- | --- |
|  |  |  |  |  |  |  |

Lipids were identified by their MS spectra resolved by MCR-ALS using the Human Metabolome Database (HMBD) (Wishart *et al*, 2012). *Lipids were detected in their protonated form (M+H)^+^, excepte TAG and DAG which were neutral and were detected as NH4^+^ adducts. In the more abundant lipìds, apart from m/z [M+H]^+^, m/z ions from the isotopic cluster were detected* (noted as M+NH4+H or M+(2*H) . One of the advantages of the non-targeted analysis approach is that it has allowed the detection of the TAG species (TAG 46:3-6, TAG 47:1-2, TAG 47:4-5, TAG 49:2-5, TAG 49:7, TAG 51:2, TAG 51:4, TAG 51:5, TAG 51:7, TAG 53:7) with lípid acids with an even number of C atoms, probably coming from the D.magna diet.

Lipids were identified by their MS spectra resolved by MCR-ALS using the Human Metabolome Database (HMBD) (Wishart *et al*, 2012). Lipids were detected in their protonated form (M+H)^+^, excepte TAG and DAG which were neutral and were detected as NH4^+^ adducts. In the more abundant lipìds, apart from m/z [M+H]^+^, also the isotòpic M^+2^ was detected. One of the advantages of the non targeted analysis approach is that it has allowed the detection of the TAG species (TAG 46:n, TAG 47:n, TAG 49:n,TAG 51:n TAG 53:n) with lípid acids with an even number of C atoms, probably coming from the D.magna diet.


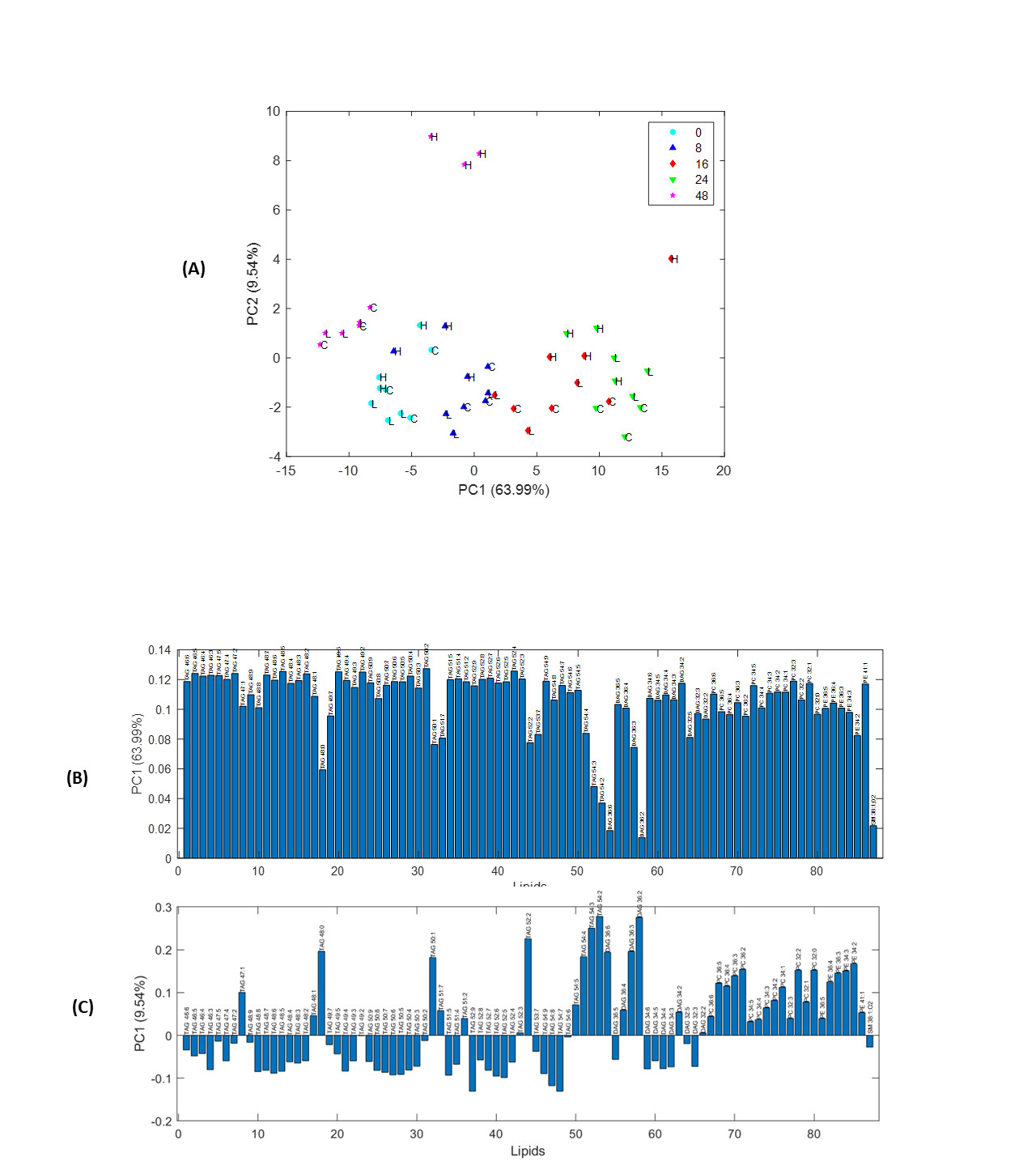


**Figure S4.** PCA plots of the peak areas of the MCR-ALS resolved elution profiles (A) PCA scores plot, (B) First component (PC1) loadings plot of and (C) Second component (PC2) loadings plot


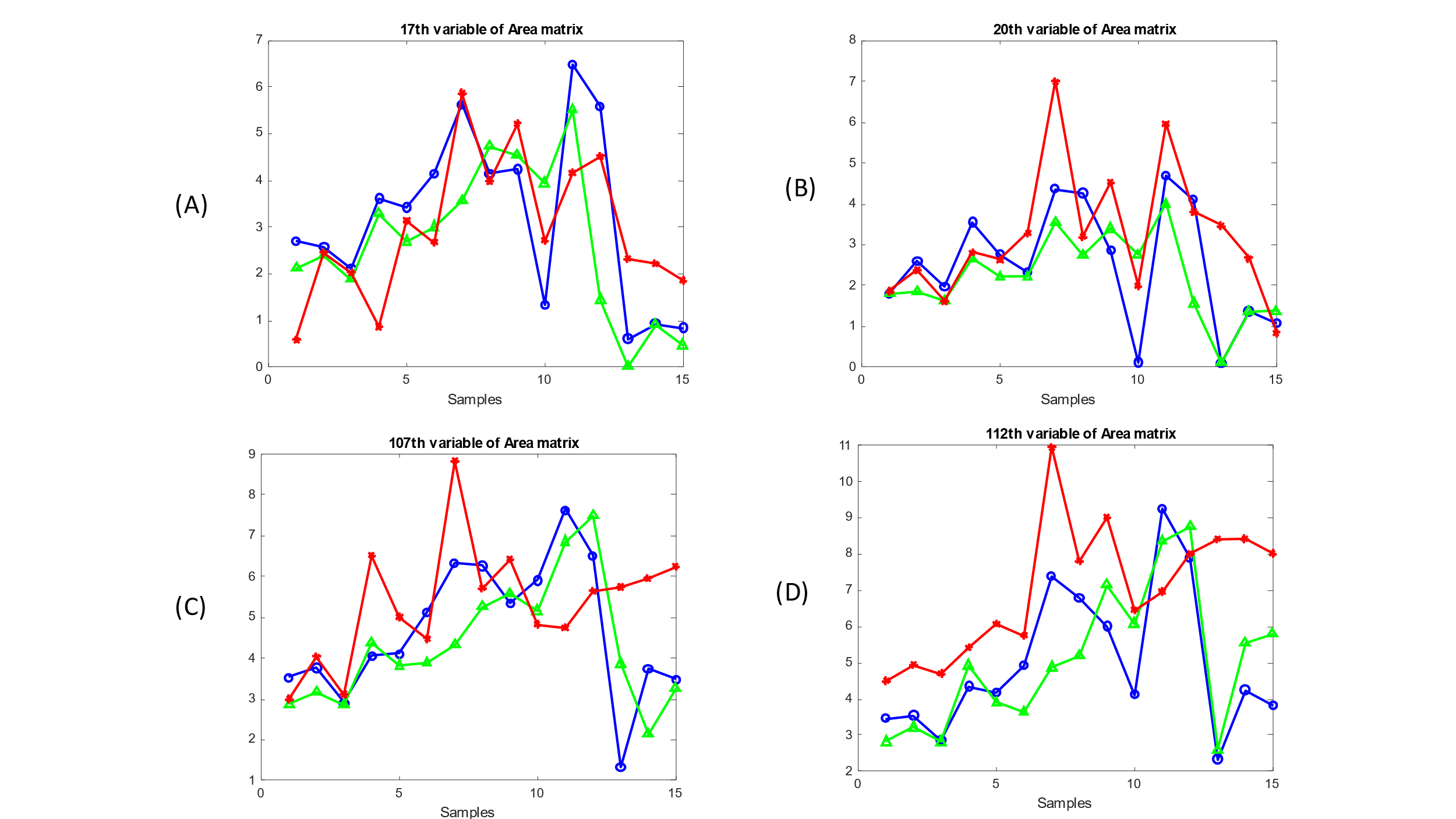


**Figure S5.** Interaction plots of the changes in the concentrations of the lipids (see Supporting Table 1) (A) TAG 48:4 (B) TAG 48:1 (C) PC 34:2 and (D) PC 32:2

If the resulting effect of the two factors is equal to the product of their effects when they act alone, the combined effect is considered to be a multiplicative effect in which the effect of one factor depends on the magnitude of the other factor in a multiplicative manner. A synergistic effect occurs when the combined effect of the two factors is greater than the sum of their individual effects (additive effect). A type of synergistic effect occurs when one factor enhances the effect of another factor that has little or no activity on its own. This is also called a potentiating effect. An antagonistic effect, on the other hand, occurs when the combined effect of the two factors is less than the sum of their individual effects. Finally, we define an unknown, variable effect, as one that does not follow a particular trend (no additive, non-multiplicative…).


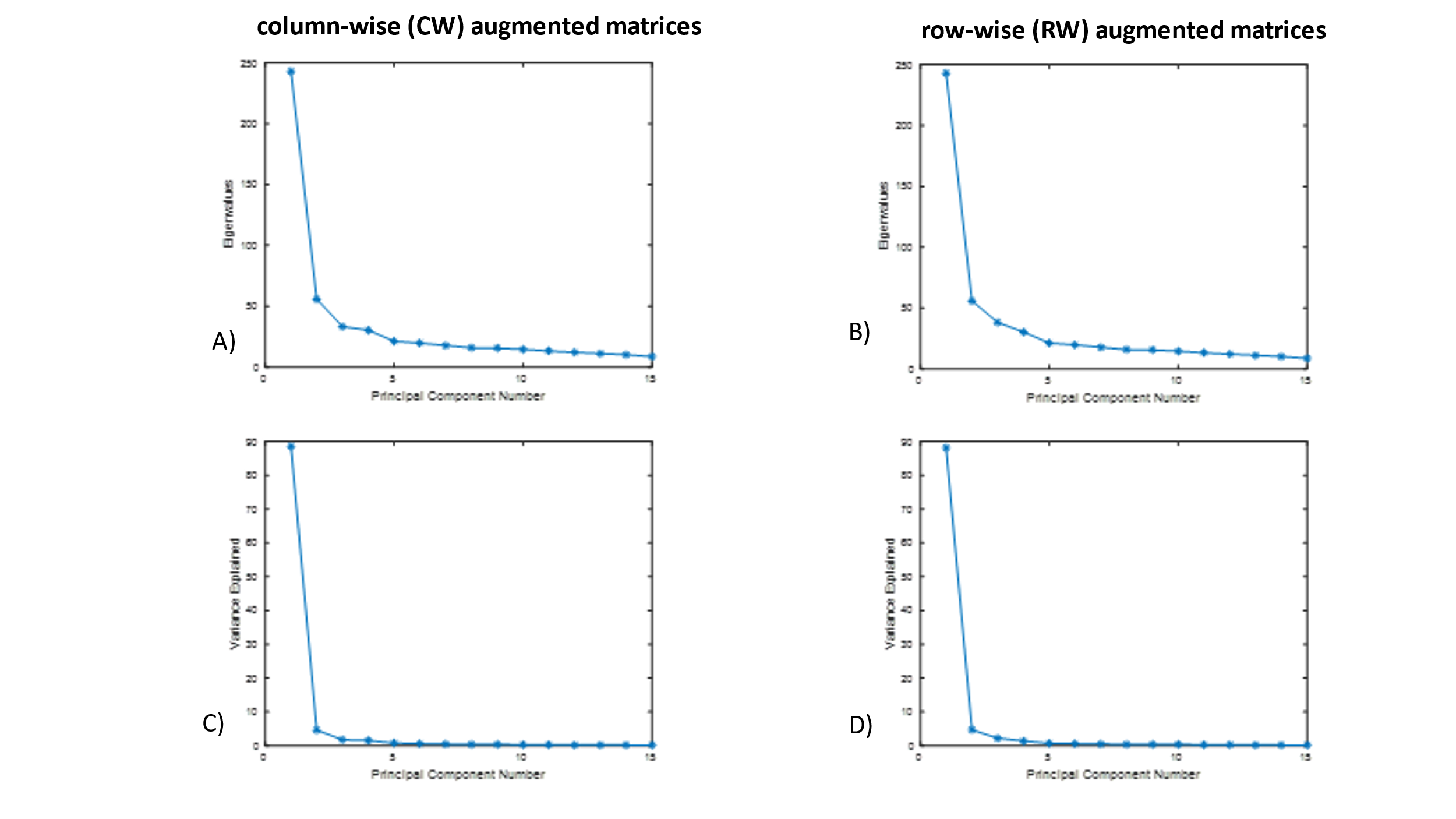


**Figure S6.** Singular Value decomposition (SVD) of the peak areas of the elution profiles data matrices of the 45 samples under the three TBT concentrations (control, low and high) arranged by A) vertical concatenation (column-wise augmentation, CW) or B) horizontal concatenation (row-wise augmentation, RW). Plot of the explained variances by the principal components of the (C) column-wise (CW) and (D) row-wise (RW) augmented peak area data matrices.

Three-way datasets can be reshaped in three possible two-way augmented data matrices based on their three different data modes (ways or directions). Individual two-way data matrices can be vertically concatenated with a column-wise augmented data matrix and horizontally with a row-wise augmented data matrix. As shown in Figure S5A and S5B, the SVD of column and row-wise augmented data matrix will give a similar number of significant singular values (four larger singular values; the other singular values are associated with experimental noise), which will be equal to the number of chemical sources of data variance in the analyzed peak areas data matrices. If a trilinear model is suitable for the analysis of a three-way data set and there is no degeneracy, rank overlap, nor rank deficiency in any of the three modes (De Juan & Tauler, 2001; Tauler, 2021; Tauler *et al,* 1995), the application of Singular Value Decomposition, SVD, (Klema & Laub, 1980)) produces the same number of very large singular values ​​in the two cases compared to those associated with experimental noise (Zhang & Tauler, 2022). This is often a simple test useful for examining if the three-way data structures are suitable for their analysis by a trilinear factor decomposition model. Based on Figures S5C and S5C, the first four components explained more than 99% of the variance in both, the column and row-wise augmented data matrices.

**References**

De Juan A, Jaumot J, Tauler R (2014) Multivariate Curve Resolution (MCR). Solving the mixture analysis problem. *Analytical Methods* 6: 4964-4976. doi: [10.1039/C4AY00571F](https://doi.org/10.1039/C4AY00571F)

De Juan A, Tauler R (2001) Comparison of three‐way resolution methods for non‐trilinear chemical data sets. *Journal of chemometrics* 15: 749-771. doi: [10.1002/cem.662](http://dx.doi.org/10.1002/cem.662)

Gorrochategui E, Jaumot J, Tauler R (2019) ROIMCR: a powerful analysis strategy for LC-MS metabolomic datasets. *BMC bioinformatics* 20: 1-17. doi: [10.1186/s12859-019-2848-8](https://doi.org/10.1186/s12859-019-2848-8)

Jaumot J, de Juan A, Tauler R (2015) MCR-ALS GUI 2.0: New features and applications. *Chemometrics and Intelligent Laboratory Systems* 140: 1-12. doi: [10.1016/j.chemolab.2014.10.003](https://doi.org/10.1016/j.chemolab.2014.10.003)

Klema V, Laub A (1980) The singular value decomposition: Its computation and some applications. *IEEE Transactions on automatic control* 25: 164-176. doi: [10.1109/TAC.1980.1102314](https://doi.org/10.1109/TAC.1980.1102314)

Tauler R (1995) Multivariate curve resolution applied to second order data. *Chemometrics and intelligent laboratory systems* 30: 133-146. doi: [10.1016/0169-7439(95)00047-X](https://doi.org/10.1016/0169-7439(95)00047-X)

Tauler R (2021) Multivariate curve resolution of multiway data using the multilinearity constraint. *Journal of Chemometrics* 35: e3279. doi: [10.1002/cem.3279](https://doi.org/10.1002/cem.3279)

Tauler R, Smilde A, Kowalski B (1995) Selectivity, local rank, three‐way data analysis and ambiguity in multivariate curve resolution. *Journal of Chemometrics* 9: 31-58. doi: [10.1016/0169-7439(95)00047-X](https://doi.org/10.1016/0169-7439(95)00047-X)

Zhang X, Tauler R (2022) Flexible Implementation of the Trilinearity Constraint in Multivariate Curve Resolution Alternating Least Squares (MCR-ALS) of Chromatographic and Other Type of Data. *Molecules* 27: 2338
